# Ensuring Safe Surgery: A Closed-Loop Audit of the WHO Surgical Safety Checklist Practices

**DOI:** 10.7759/cureus.85868

**Published:** 2025-06-12

**Authors:** Mohammad Taha Kamal, Uzma Shamim Seth, Bilal Fattani, Muhammad Taha Junaid Khan, Sadia Lateef, Mohammad F Umer

**Affiliations:** 1 Surgery and Allied, Jinnah Medical and Dental College, Karachi, PAK

**Keywords:** compliance, patient safety, perioperative care, surgical safety, who checklist

## Abstract

Background and aim: The World Health Organization (WHO) developed the Surgical Safety Checklist (SSC) to support critical safety steps during surgeries. Although these standards were endorsed worldwide, many healthcare settings were not following them properly. This study aimed to assess the adherence to the WHO Surgical Safety Checklist at Sohail Trust Hospital, Karachi, and to identify which safety steps were often neglected during surgical procedures.

Methods: A prospective observational study was conducted from December 2024 to February 2025 at Sohail Trust Hospital, Karachi, under ethical approval #00126/25. A total of 120 surgical procedures were observed using a consecutive sampling technique. Compliance with each of the 18 recommended SSC items was recorded based on WHO guidelines. Data were collected across the following three surgical phases: before induction of anesthesia, before skin incision, and before the patient left the operating room. Descriptive statistics were used to summarize the data using SPSS version 26.0 (Armonk, NY: IBM Corp.), released in 2019.

Results: A total of 120 surgical procedures were audited, with 60 cases each in the pre- and post-intervention phases. Overall checklist compliance improved from 58.36% to 78.05% following an educational intervention (p=0.015, Wilcoxon signed-rank test). The greatest improvement was noted in antibiotic prophylaxis (0%-85%), while compliance for blood preparation in high-risk cases declined. Procedure-wise, hernioplasty and appendectomy showed the highest gains in adherence. The intervention demonstrated a large effect size (Cohen’s d=0.61).

Conclusion: A simple educational intervention significantly enhanced adherence to the WHO Surgical Safety Checklist, particularly in elective surgeries. These findings support the value of targeted training and continuous monitoring to improve surgical safety. Persistent gaps in emergency preparedness highlight the need for tailored strategies. Sustained efforts and team-based communication are essential to achieve long-term compliance and safer surgical outcomes.

## Introduction

Safety in surgery ensures the quality of healthcare delivery, particularly in lower and middle-income countries (LMICs) [1]. Where systematic limitations undermine the patient outcomes. Despite progress in surgical tools and methods, mistakes by medical staff still result in problems during surgery [2]. To address this problem, the World Health Organization (WHO) developed the Surgical Safety Checklist (SSC) as a worldwide intervention to reduce surgical risks and improve the way the surgical team interacts during surgery and its phases [3].

In SSC evaluation, it is important to review three steps as follows: checking in (before anesthesia is given), checking out before the incision is made to the skin (time-out), and checking out for the last time as the patient leaves the room (sign-out) [4]. Structured reviews during surgical rounds help the SSC to standardize surgery, bring the team closer together, and guarantee that the necessary safety steps have been achieved in each case [5]. Despite the SSC’s achievements in various countries and settings, its real-world applicability is varied [6]. A number of international reports have demonstrated that many institutions from different regions have inconsistent adherence to SSC practices, which is usually linked to less formal training of staff, local culture of the institution, and access to resources [7,8].

In Pakistan, information on how well hospitals follow surgical safety guidelines was scarce, particularly in the private sector, which made it necessary to explore the matter. This study aimed to assess the compliance of the SSC provided by the WHO in Sohail Trust Hospital, a major healthcare center in Karachi. Through the analysis of adherence in all three phases for a given number of operations, the study pointed out the weaknesses in implementation and suggested possible improvements. The conclusions helped to plan better training for surgeons and introduced improved policies for safer surgical care.

## Materials and methods

This prospective, observational, closed-loop clinical audit was conducted at Sohail Trust Hospital, a tertiary care facility affiliated with Jinnah Medical and Dental College (JMDC), Karachi, between December 2024 and February 2025. Ethical approval was obtained from the Ethical Review Committee, Jinnah Medical and Dental College (#00126/25). A total of 120 surgical procedures were audited across two cycles, with 60 surgeries observed in each phase using consecutive sampling. The audit tool was based on the original WHO Surgical Safety Checklist, which consists of 19 items across three perioperative phases as follows: sign in (before induction of anesthesia), time out (before skin incision), and sign out (before the patient leaves the operating room) [9]. Compliance with each item was assessed by trained auditors who observed whether each component was verbalized and/or performed during surgery. A structured data collection form was used to document compliance, along with basic patient demographics and procedure type.

Auditors received formal training on the use of the checklist to ensure standardized observations before data collection. The first audit cycle was conducted from December 1 to December 30, 2024. And after this, an educational intervention was implemented on December 30, 2024. It included a departmental presentation on the importance of checklist adherence, dissemination of first-cycle findings, and distribution of official circulars signed by the department head mandating checklist use. Similarly, the second audit cycle was carried out by following the same protocols from January 13 to February 15, 2025. At the end of the second cycle, a structured interview form was used to gather qualitative information about barriers like feasibility, awareness, training, and motivation. Data were analyzed using SPSS version 26.0 (Armonk, NY: IBM Corp.), released in 2019. Descriptive statistics were calculated for checklist compliance rates. A Wilcoxon signed-rank test was performed to determine whether the change in compliance rate between the two audit phases was statistically significant or not, using a p<0.05.

## Results

A total of 120 surgeries were included in this audit as follows: 60 procedures in the pre-intervention phase (cycle 1) and 60 in the post-intervention phase (cycle 2). The observed surgeries comprised a variety of procedures, including laparoscopic cholecystectomy, appendectomy, hernioplasty, laparotomy, and examination under anesthesia (EUA). Each surgery was evaluated for compliance with the World Health Organization (WHO) Surgical Safety Checklist across its three defined phases as follows: sign in, time out, and sign out. Results from the Wilcoxon signed-rank test indicated that a statistically significant change toward better checklist compliance occurred when the intervention was performed (test statistic=17.0, p=0.015). The results also indicated a significant impact, as the effect size measured by Cohen’s d was 0.61.

Assessment of compliance determined whether the individual checklist requirements were being met. Adherence rates improved significantly for nearly all items on the checklist once the educational intervention was introduced (Table 1).

**Table 1:** Comparison of the WHO Surgical Safety Checklist compliance across two audit cycles. Values represent the percentage of surgeries where each checklist item was compliant. Wilcoxon signed-rank test, p=0.015 (p<0.05 was considered significant).

Checklist item	Cycle 1, n (%)	Cycle 2, n (%)
Sign in		
Identity confirmed by anesthesia	60 (100.0%)	60 (100.0%)
Patient asked about allergies	60 (100.0%)	60 (100.0%)
Site marked	21 (35.7%)	60 (100.0%)
Airway assessment performed	60 (100.0%)	60 (100.0%)
Anesthesia equipment checked	45 (75.0%)	46 (76.7%)
Blood arranged (massive loss risk)	24 (40.0%)	0 (0.0%)
IV access secured (double/central line)	24 (40.0%)	0 (0.0%)
Time out		
Antibiotic prophylaxis within 60 minutes	0 (0.0%)	51 (85.0%)
Patient-specific concerns (anesthesia)	35 (58.3%)	39 (65.0%)
Critical steps communicated (surgeon)	47 (78.3%)	56 (93.3%)
Surgery duration estimated (surgeon)	31 (51.7%)	55 (91.7%)
Anticipated blood loss discussed (surgeon)	42 (70.0%)	55 (91.7%)
Name confirmed by all team members	55 (91.7%)	57 (95.0%)
Sign out		
Equipment issues confirmed by nurse	14 (23.3%)	41 (68.3%)
Instrument count confirmed by nurse	53 (88.3%)	57 (95.0%)
Procedure name confirmed	60 (100.0%)	60 (100.0%)
Specimen labeled and sent	15 (25.7%)	46 (77.1%)
Recovery concerns confirmed (surgeon)	18 (30.0%)	49 (81.7%)
Average compliance per phase		
Sign in phase	42 (70.1%)	52 (86.1%)
Time out phase	35 (58.3%)	52 (86.9%)
Sign out phase	32 (53.5%)	51 (84.4%)
Overall compliance	35 (58.3%)	47 (78.1%)

The greatest improvement was seen in the proper and timely use of antibiotic prophylaxis, which increased from 0 (0%) to over 51 (85.0%). On the other hand, blood preparation for massive blood loss showed no improvement, and in fact, it went down from 24 (40.0%) to 0 (0%). Before the intervention, only 35 (58.3%) of checklist items were followed, but that increased to 47 (78.1%) afterward, showing that compliance rose by 19.69%. Table 2 shows the total number of checklist items completed and compared across different types of procedures observed in both audit cycles.

**Table 2:** Comparison of overall surgical safety checklist compliance by procedure type (cycle 1 vs. cycle 2). Compliance scores reflect mean adherence across all checklist phases. Sign "+" shows improvement and "-" shows decline.

Surgical procedure	Cycle 1 compliance (%)	Cycle 2 compliance (%)	Absolute change (%)
Laparoscopic cholecystectomy	70.7%	92.2%	+21.5%
Appendectomy	66.3%	89.3%	+23.0%
Hernioplasty	54.4%	88.3%	+33.9%
Examination under anesthesia (EUA)	63.9%	80.4%	+16.5%
Laparotomy	38.9%	33.3%	-5.6%

Increases were observed in hernioplasty (+33.9%) and appendectomy (+23.0%), as both surgeries are commonly performed in organized healthcare settings. Alternatively, in emergencies where laparotomy was performed, adherence to the checklist dropped slightly (-5.6%), possibly because teams found it difficult to stick to the checklist in such stressful conditions.

## Discussion

The findings of this audit made it clear that structured educational intervention could bring about significant improvements in compliance rate, causing an overall adherence to rise from 35 (58.3%) to 47 (78.05%). Similar to earlier studies, the findings from this study demonstrated that education on this subject was a critical factor for surgeons and teams to adhere to surgical checklists [10,11]. Before the intervention, compliance for identifying the patient and checking for allergic diseases was high and stayed at 60 (100%) in both audit cycles. However, performance was reduced for steps that required plenty of communication, like giving antibiotics, estimation of blood loss, and discussion of recovery plans. The pattern was similar to the findings from international literature, where verbal communication was often related to differences in ranks, lack of training, or time constraints [12,13].

As a result of the educational session, compliance improved a lot on most checklist items, mainly among domains where it had been low. The percentage of patients who received prophylactic antibiotics promptly went from 0 (0%) to 51 (85.0%). The improvement was likely due to increased attention and practical reinforcement given to these guidelines during the intervention. Also, improvements in site marking, explanation of critically important steps, and review upon blood loss demonstrated that interdisciplinary communication and awareness had increased among surgical teams. Studies showed better results with compliance when communication among team members was good [14,15].

However, specific areas kept showing continuous gaps. There was a decrease from 24 (40%) to 0 (0%) compliance for blood products and setting up access lines in surgeries that predict significant bleeding. Although the decline was not large as a whole but steps were significant, particularly for surgeons to take care of, and might be caused by differences in how staff interpreted the information. Some studies aligned with the fact that skipping these important steps or overlooking them had caused some serious problems during surgeries [16,17]. Furthermore, patients experienced lower compliance in laparotomy cases, emphasizing the difficulties of carrying out checklists during emergencies. Such circumstances usually shorten the decision-making window and may prevent everyone from participating consistently in the checklist [18].

There were several limitations that needed to be addressed. Since the audit was conducted at only one location, the results could not be generalized to all situations. Even though the observational design could add in subjective views, steps were taken to control bias through the training of auditors and blinding of teams. Besides, the long-term stability of improvements in compliance was not examined as part of the study. As a result of this study, it became clear that planned training in hospitals could increase the rate of compliance with the WHO Surgical Safety Checklist prior to elective surgeries.

## Conclusions

As a result of the closed-loop audit, it was determined that providing a structured approach to education helped in making surgery safer. There were good improvements in antibiotic administration and how teams communicated. Procedures such as hernioplasty and appendectomy showed the most improvement, proving that the checklist compliance was good during controlled operations. Moreover, the effect size of the intervention demonstrated that it was useful for both research and practical use.

Still, particular areas within emergency preparedness, such as organizing blood and setting up intravenous injections (IVs), pointed towards major gaps. In the future, attention should be given to designing specific strategies for emergencies and to causing continuous changes in culture for improvements. Encouraging both accountability and teamwork from different areas will continue to play a major role in long-term progress.
